# Genomic Evolution of *Staphylococcus aureus* During Artificial and Natural Colonization of the Human Nose

**DOI:** 10.3389/fmicb.2019.01525

**Published:** 2019-07-05

**Authors:** Manisha Goyal, Fabien Javerliat, Mattia Palmieri, Caroline Mirande, Willem van Wamel, Mehri Tavakol, Nelianne J. Verkaik, Alex van Belkum

**Affiliations:** ^1^Data Analytics Unit, bioMérieux, La Balme-les-Grottes, France; ^2^Microbiology R&D, bioMérieux, La Balme-les-Grottes, France; ^3^Department of Medical Microbiology and Infectious Diseases, Erasmus University Medical Center, Rotterdam, Netherlands

**Keywords:** *Staphylococcus aureus*, nasal carriage, epidemiology, resistance, MLST, SNP, strain relatedness, genomic evolution

## Abstract

*Staphylococcus aureus* can colonize the human vestibulum nasi for many years. It is unknown whether and, how *S. aureus* adapts to this ecological niche during colonization. We determined the short (1 and 3 months) and mid-term (36 months) genomic evolution of *S. aureus* in natural carriers and artificially colonized volunteers. Eighty-five *S. aureus* strains were collected from 6 natural carriers during 3 years and 6 artificially colonized volunteers during 1 month. Multi-locus sequence typing (MLST) and single nucleotide polymorphism (SNP) analysis based on whole-genome sequencing (WGS) were carried out. Mutation frequencies within resident bacterial populations over time were quantified using core genome SNP counts (comparing groups of genomes) and pairwise SNP divergence assessment (comparing two genomes from strains originating from one host and sharing identical MLST). SNP counts (within 1–3 months) in all naturally colonizing strains varied from 0 to 757 (median 4). These strains showed random and independent patterns of pairwise SNP divergence (0 to 44 SNPs, median 7). When the different core genome SNP counts over a period of 3 years were considered, the median SNP count was 4 (range 0–26). Host-specific pairwise SNP divergence for the same period ranged from 9 to 57 SNPs (median 20). During short term artificial colonization the mutation frequency was even lower (0–7 SNPs, median 2) and the pairwise SNP distances were 0 to 5 SNPs (median 2). Quantifying mutation frequencies is important for the longitudinal follow-up of epidemics of infections and outbreak management. Random pattern of pairwise SNP divergence between the strains isolated from single carriers suggested that the WGS of multiple colonies is necessary in this context. Over periods up to 3 years, maximum median core genome SNP counts and SNP divergence for the strains studied were 4 and 20 SNPs or lower. During artificial colonization, where median core genome SNP and pairwise SNP distance scores were 2, there is no early stage selection of different genotypes. Therefore, we suggest an epidemiological cut off value of 20 SNPs as a marker of *S. aureus* strain identity during studies on nasal colonization and also outbreaks of infection.

## Introduction

Extensive use of antibiotics in the environment and the clinical domain contributes toward the emergence of (multi-)drug resistant bacterial pathogens. This has become a global threat (Roca et al., 2015). *Staphylococcus aureus* (*S. aureus*) is among the bacterial species associated with increasing drug resistance, morbidity, invasive disease, and mortality in humans as well as animals (Chambers and Deleo, 2009; Schmidt et al., 2015; Li and Webster, 2018). *S. aureus* is a common opportunistic human pathogen identified most often on the nasal epithelium, About 30–50% of healthy individuals are persistently colonized (Wertheim et al., 2005). *S. aureus* causes a large variety of community as well as hospital-acquired infections. These include deep abscesses, endocarditis, osteomyelitis, pneumonia, and bloodstream infections (Foster and Höök, 1998; Rasigade and Vandenesch, 2014; Taylor and Unakal, 2018). *S. aureus* nasal carriage is a risk factor for the development of staphylococcal infections. Adherence to the human nasal epithelial cells is a prerequisite for *S. aureus* colonization and initiation of infection (Roche et al., 2003). The prevalence of non-symptomatic colonization with methicillin resistant *S. aureus* strains in the open United States population escalated from 0.8 to 1.5% over recent years (Gorwitz et al., 2008).

Colonization begins with the interaction between nasal epithelial ligands and bacterial receptors often cataloged as microbial surface components recognizing adhesive matrix molecules (MSCRAMMS) (Foster and Höök, 1998; Ghasemian et al., 2015). During colonization *S. aureus* expresses adherence genes (*clf* B, *isd*A, *fnb*A, *eap, sce*D, *oat*A, and *atl*A) and several immune-modulating genes (e.g., *sak, chp, spa*, and *scn*) (Burian et al., 2010; Baur et al., 2014). Host factors and local microbiota can affect the adhesion and colonization properties of *S. aureus* as well (Emonts et al., 2007; Frank et al., 2010; Ruimy et al., 2010).

During colonization, *S. aureus* secretes a number of immune-modulating proteins. Staphylococcal complement inhibitor (SCIN), encoded by the *scn* gene, can efficiently protect *S. aureus* by inhibiting the innate immune response mediated by human neutrophils. SCIN and other immune modulating proteins are encoded on the immune evasion cluster (IEC) (Goerke et al., 2006). The *scn* gene was identified as a conserved one being present in all IEC (van Wamel et al., 2006). To test the role and stability of IEC human artificial inoculation was performed using isolates with and without IEC. It was concluded that IEC may not play a significant role in adherence but it did display an essential role in propagation and long term survival (Verkaik et al., 2011).

We have here used whole genome sequencing (WGS) to quantify the mutational changes occurring in *S. aureus* strains during natural and artificial nasal colonization during periods ranging between 1 and 36 months. The numbers of human volunteers and hence the overall number of *S. aureus* nasal isolates are limited due to the technical and logistic complexity of the studies involved (Verkaik et al., 2011). In addition, studies involving colonization of human volunteers have to follow extensive ethical procedures and protocols. We applied bio-informatics approaches to assign MLST types and to detect genetic variation at the single nucleotide polymorphism (SNP) level. Moreover, we analyzed selective presence of virulence factors for all strains.

## Materials and Methods

### Description of the Strain Collection

*Staphylococcus aureus* strain collection was carried out as described earlier (Verkaik et al., 2011) at Erasmus Medical Center (Rotterdam, Netherlands). Naturally colonizing strains were isolated from nasal swab cultures from healthy persistent carriers who were positive for *S. aureus* at five culture moments over a time interval of 3 months in both 2007 and 2010. Artificially colonizing strains were collected from the human volunteers inoculated with *S. aureus* strain NCTC 8325-4 with or without IEC and follow-up cultures were performed in 2008 (days 1, 2, 3, 4, 7, 14, 21, and 28 after inoculation). The latter strains were susceptible to all common antibiotics and were free from staphylococcal toxin genes (Williams et al., 1997; Wertheim et al., 2008). A review of all strains sequenced is provided in Supplementary Table 1.

### *S. aureus* Genome Sequences

Isolates were sequenced by WGS (Illumina HiSeq 2000 platform). Raw reads were assembled using the A5 MiSeq-20140604 assembler. Datasets for strains cultured in 2007 and 2010 contained 35 and 22 isolates, respectively, involving natural nasal colonization in 6 persistent carriers. The dataset from 2008 (28 isolates) was collected for strains from 6 different volunteers artificially colonized with *S. aureus* strain NCTC 8325-4. DNA isolation was performed for up to three colonies from each culture to define their genotypic stability at different point of times during short term as well longer term nasal carriage (see Supplementary Table 1). The sequences obtained from the 1 to 3 independent colonies taken for some of the individual strains were analyzed independently by the bioinformatics tools applied. Using bioinformatics tools as BioNumerics (Applied Maths, bioMérieux, Belgium), kSNP3 (Computations/Global Security, Lawrence Livermore National Laboratory, Livermore, CA, United States and Bellingham Research Institute, Bellingham, WA, United States), and Abricate (Torsten Seemann, University of Melbourne, Australia), all genomes were analyzed extensively.

### MLST Typing and SNP Detection

To understand the genetic diversity of all the isolates multi-locus sequence typing (MLST) was performed using BioNumerics^1^. The MLST method is known to have a higher discriminatory power for *S. aureus* strains than PFGE (Peacock et al., 2002a). For classical MLST typing seven housekeeping genes and their various alleles were used to define strain relatedness^2^ (Jolley et al., 2018). A phylogenetic tree was constructed by executing the Linux based stand-alone source code of kSNP3 (Gardner et al., 2015), which identified core genome SNP counts and provided a consensus parsimony phylogenetic tree. The kmer size was set to 19, the optimum size estimated by the kSNP3 utility program Kchooser (Gardner et al., 2015). Pairwise SNP distances between later stage isolates as compared to early stage isolates from each individual were calculated to define mutation over time. The python script kSNPdist was used to calculate the pairwise SNP divergence between all *S. aureus* isolates. kSNP3 and kSNPdist executables for OS X and Linux are freely available at https://sourceforge.net/projects/ksnp/.

### Resistance and Virulence Gene Identification

All the genomes were screened for the presence of 40 known and putative virulence genes^3^ (Shukla et al., 2010) (enterotoxin genes, exotoxin genes, leucocidin genes, hemolysin genes, surface protein genes, and putative virulence genes) and the *S. aureus* antibiotic resistance genes available in the ResFinder database^4^. Those 40 genes are grouped as classical staphylococcal. The Linux-based command line tool known as Abricate was downloaded^5^ to perform additional mass screening for antimicrobial resistance or virulence genes. All the identified resistance and virulence genes in the dataset were summarized in Supplementary Table 3. Additionally, *in silico*-based mapping of the *scn* gene using BioNumerics was carried out to determine the presence of IEC (van Wamel et al., 2006).

## Results

### Quality Testing of Genome Datasets

**FIGURE 1:**
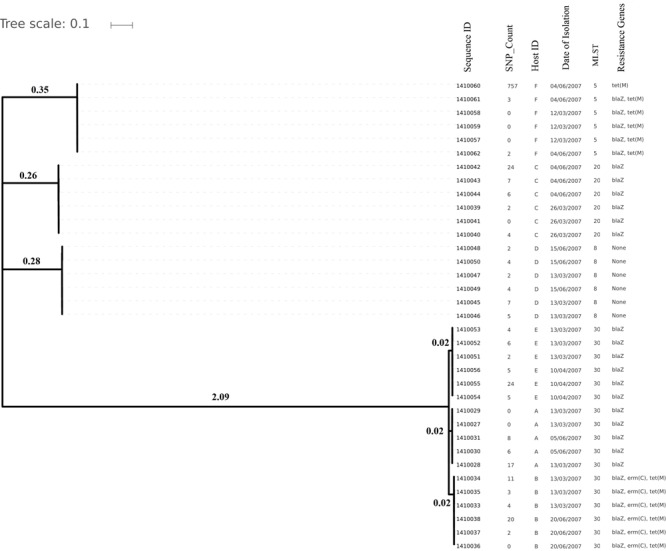
Phylogenetic tree depicting clustering on the basis of core SNP count ranges from 0 to 757 SNPs (median 4 SNPs) in all the *Staphylococcus aureus* strains colonized during 3 months (2007 subgroup) of follow up along with their date of isolation, persistent carriers from which they have isolated after maximum three cultural moments, their sequence type and resistance genes. Note that all isolates are clustered together on the basis of the original individual they were cultured from.

**FIGURE 2:**
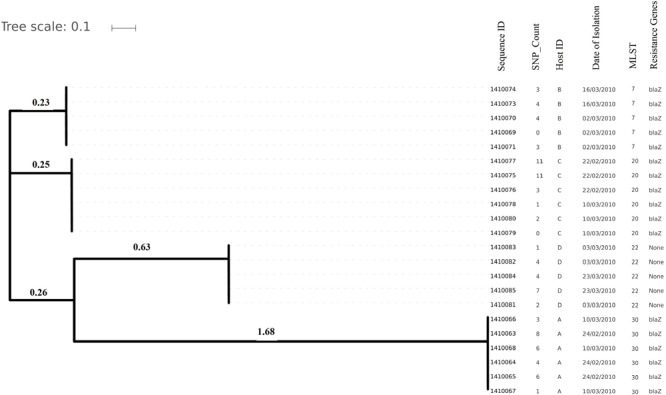
Evolutionary relationship on the basis of core genome SNP counts detected (range 0 to 11 SNPs) in *S. aureus* strains colonized and isolated during 1 month (2010 subgroup) along with the date of their isolation, the host from which they have isolated, MLST and resistance genotype. Isolates from the same host are clustered together showing their higher strain relatedness.

Genome sizes varied from 2,647 to 2,827 Kilo base (kb). The average number of contigs generated per genome was 64 contigs (ranging from 40 to 315 contigs). The average N50 contig length was 171778 bp (Supplementary Table 2). Isolates (and hence their genomes) from a single individual are expected to be part of a single clade as predicted by the MLST data and phylogenetic clustering (Figure 1, 2).

### Short Term Evolution (1–3 Months) in Naturally Colonizing *S. aureus* Strains

*Staphylococcus aureus* isolates from 2007 and 2010 (35 and 22 genomes, respectively) from carriers A to F were analyzed for short term genomic changes, over 3 months (in 2007), and 1 month (in 2010). ST30 (2007: 47%; 2010: 30%) and ST20 (2007: 17.60%; 2010: 30%) were found to be the dominant MLST types followed by ST8 and ST5 (18% each) in 2007 and ST22 and ST7 (20% each) in the 2010 subgroups (Table 1). Over the period of 3 years some of the strains were replaced by different sequence type strains within a same carrier. For instance isolates from carrier B and D in 2007 were ST30 and ST8 but in 2010, isolates from the same carries were ST7 and ST22, respectively. These strains were not included for longer term pairwise SNP divergence analysis (Table 1).

**Table 1:** Pairwise SNP distances identified between all the early and later stages isolates (according to their isolation date) among the *S. aureus* strains of subgroup 2007 and 2010 independently from each persistent nasal carrier.

	2007				2010			

Persistent carrier ID	Begin (Seq. ID)	Isolate after 3 months (Seq. ID)	No. of SNP differences	MLST	Begin (Seq. ID)	Isolate after 1 month (Seq. ID)	SNP differences	MLST
A	1410027	1410030	10	30	1410063	1410066	4	30
		1410031	11			1410067	5	
		–	–			1410068	7	
	1410028	1410030	11		1410064	1410066	4	
		1410031	10			1410067	3	
		–	–			1410068	3	
	1410029	1410030	11		1410065	1410066	3	
		1410031	13			1410067	3	
		–	–			1410068	3	
B	1410033	1410036	7	30	1410069	1410073	11	7
		1410037	7			1410074	12	
		1410038	10			–	–	
	1410034	1410036	17		1410070	1410073	10	
		1410037	17			1410074	11	
		1410038	20			–	–	
	1410035	1410036	7		1410071	1410073	13	
		1410037	7			1410074	14	
		1410038	10			–	–	
C	1410039	1410042	43	20	1410075	1410078	7	20
		1410043	16			1410079	7	
		1410044	16			1410080	9	
	1410040	1410042	44		1410076	1410078	3	
		1410043	18			1410079	2	
		1410044	18			1410080	2	
	1410041	1410042	43		1410077	1410078	8	
		1410043	15			1410079	7	
		1410044	15			1410080	8	
D	1410045	1410048	25	8	1410081	1410084	2	22
		1410049	3			1410085	3	
		1410050	26		1410082	1410084	1	
	1410046	1410048	27			1410085	4	
		1410049	3		1410083	1410084	2	
		1410050	27			1410085	4	
	1410047	1410048	24	
		1410049	3	
		1410050	26	
E	1410051	1410054	2	30
		1410055	1	
		1410056	0	
	1410052	1410054	6	
		1410055	22	
		1410056	5	
	1410053	1410054	5	
		1410055	22	
		1410056	3	
F	1410057	1410060	9	5
		1410061	2	
		1410062	0	
	1410058	1410060	9	
		1410061	2	
		1410062	0	
	1410059	1410060	8	
		1410061	2	
		1410062	0	

*Multilocus sequence typing was assigned to all the isolates of each host. From one carrier, multiple colonies were selected and sequenced to count the number of SNP differences between different colonies. Exceptionally from carrier A strain id 1410032 was shifted to ST22 and isolate 1410072 was contaminated thus not included in SNP analyses.*

Core genome SNP counts for the genomes of all the strains collected in 2007 and 2010 ranged from 0 to 757 SNPs (median 4 SNPs) and 0 to 11 SNPs (median 3.5 SNPs), respectively (Figure 1, 2). We observed a small pairwise SNP distance between all the early and the later stage isolates within a carrier (all carriers pooled, 2007 median number of SNP divergence was 10 and in 2010 median SNP distance was 4 (Table 1). The maximum number of pairwise SNP differences calculated for the genomes of the isolates of carrier C ranged from 15 to 44 SNPs followed by 3 to 27 SNPs in strains from carrier D, 0 to 22 in strains from E, 7 to 20 in strains from B, 10 to 13 in strains from A and 0 to 9 SNPs in strains from F (Table 1). Paired SNP differences were also calculated for strains from subgroup 2010 illustrating the highest ranges (10–14) among strains from host B followed by 2 to 9 SNPs in strains from C, 3 to 7 in strains from A, and 1 to 4 in strains from individual D (Table 1). On an individual basis, the pattern of pairwise SNP differences is relatively random between the isolates from early and later stages of colonization.

### Longer Term Evolution (3 Years) in Naturally Colonizing *S. aureus* Strains

**FIGURE 3:**
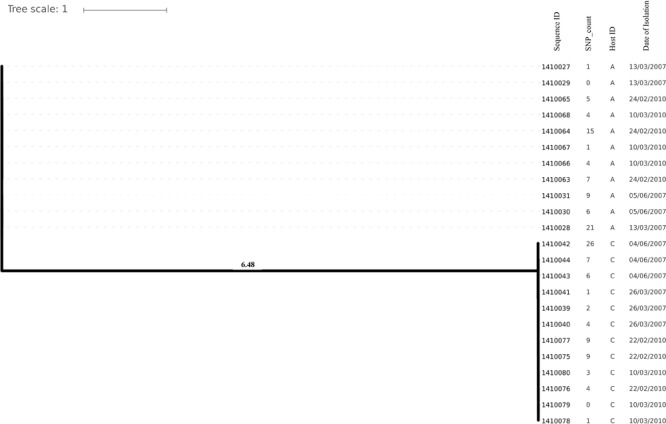
Phylogenetic tree showing longer term (3 years) diversity and relatedness of *S. aureus* strains on the bases of core genome SNP counts ranged from 0 to 26 SNPs in all the isolates from two nasal carriage individuals (A and C) for both the years 2007 and 2010.

**FIGURE 4:**
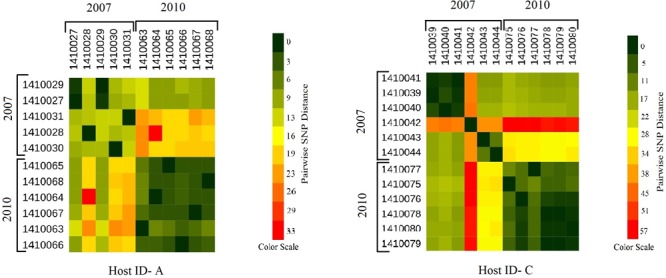
Heat maps showing the host specific pairwise SNP divergence (longer term) among all the early (2007) and later stage (2010) isolates of carrier A and carrier C individually with the color range of dark green (least SNP divergence) to red (higher SNP divergence). In both the hosts A and C pairwise SNP distances between the isolates of 2007 and 2010 datasets are visibly higher (from yellow to red boxes) than that of within the dataset itself (from dark green to light green boxes) with one exceptional isolate 1410042 in carrier C which showed higher pairwise SNP divergence within its dataset (orange boxes) as well as with the isolates of 2010 dataset (red boxes).

Evolutionary analysis over a period of 3 years (2007–2010) could only be done for the isolates from two persistent carriers, A, and C. In these carriers the MLST type remained unchanged over time, suggesting persistent colonization with the same strains (Table 1). All isolates of carrier A and C from both 2007 and 2010 were analyzed for the presence of core genome SNPs which ranged from 0 to 26 SNPs (median 4 SNPs) (Figure 3). Host specific pairwise SNP differences between the isolates dating 2007 and 2010 for both carriers A and C individually were 9–33 SNPs (median 19) and 15-57 SNPs (median 24), respectively (Figure 4). All strains from one carrier showed random distribution of SNPs; e.g., SNP distances between strains 1410027 and 1410029 versus the later stage strain 1410066 were 9 and 11 SNPs (Figure 4). This demonstrated that genomic evolution was random and none of the SNPs were fixed genetically over time.

### Mutational Analyses of Strains From Artificially Colonized Humans

**FIGURE 5:**
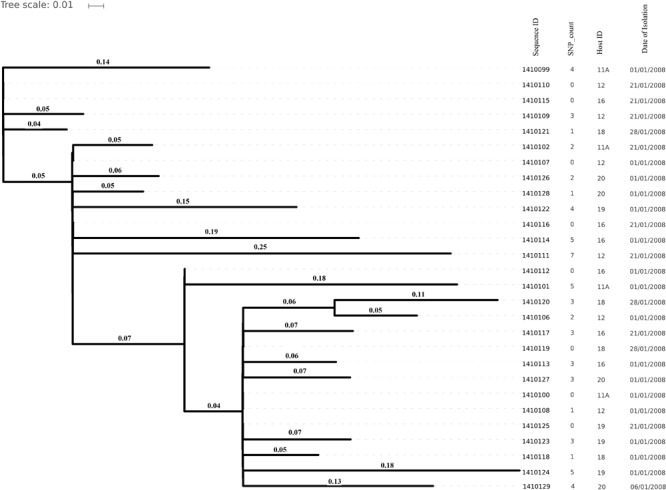
Core genome SNP counts based phylogenetic tree illustrating the close resemblance among the genomes isolated from artificially inoculated *S. aureus* nasal carriers in 2008. Core genome SNP counts here ranged from 0 to 7 core SNPs and each cluster is showing random collection of the strains irrespective of their specific host depicted very less genomic evolution (in 1 month) in artificially colonizing strains.

All strains were of ST8. No considerable identity was observed with resistance genes (Supplementary Table 3) from the database which was in agreement with the pan-susceptibility of the isolates. The overall core genome SNP counts among the isolates ranged from 0 to 7 SNPs (average 2) (Figure 5). The maximum range of pairwise SNP distances between the isolates within a host was 0 to 5 SNPs (median SNP distance 2) after 28 days of colonization in *S. aureus* nasal carriers (Table 2).

**Table 2:** Pairwise SNP distances found in artificially colonizing strains isolated during short term colonization in different individuals.

Host ID	2008 (28 days)		SNP differences
	Begin	End	
11A	1410099	1410102	4
	1410100		1
	1410101		4
12	1410106	1410109	3
		1410110	2
		1410111	5
	1410107	1410109	2
		1410110	1
		1410111	4
	1410108	1410109	2
		1410110	0
		1410111	3
16	1410112	1410115	0
		1410116	0
		1410117	0
	1410113	1410115	1
		1410116	1
		1410117	1
	1410114	1410115	3
		1410116	2
		1410117	3
18	1410118	1410119	1
		1410120	4
		1410121	2
19	1410122	1410125	2
	1410123		1
	1410124		2
20	1410126	1410129	3
	1410127		3
	1410128		3

Fourteen virulence genes (*sea, hla, hlb, hld, hlgB, clfA, clfB, fnbA, fnbB, icaA, sdrC, sdrD, sdrE*, and *tsst-1*) were identified in the current sequence dataset (Supplementary Table 3). The virulence factor *fnbA* was not found in isolates from host B and was also missing in one of the isolates from carrier F (1410060). Two strains (1410054 and 1410055) were shown to have acquired the *cna* gene during colonization of host E (Supplementary Table 3). Absence of the *scn* gene corroborating the complete lack of IEC in artificially colonized strains (Supplementary Table 3).

## Discussion

In the present work, we have studied the evolutionary patterns in nasal *S. aureus* strains to better understand their local adaptive behavior and mutational frequency. Low core genome SNP values among all the genomes defines the significant strain relatedness witnessed in this study. This is experimentally supported by the outcomes of previous research (Ankrum and Hall, 2017) where *S. aureus* strains with <71 SNP differences were considered as non-discriminate. Similar findings by Golubchik et al. (2013) suggested that SNP divergence with in a host varied from ∼ 0 to 27 SNPs among host specific isolates. In our study, one isolate from host C (1410042) was showing an exceptionally high SNP divergence value for which we have no clear explanation (Figure 5 and Table 1). Phylogenetic trees (Figure 1, 2, 3, 5) were constructed on the basis of core genome SNPs identified within strains from all individual hosts showing different numbers of mutations as compared to their pairwise frequency of SNP divergence. The level of diversity (SNP divergence) within the hosts was consistently lower than that detected between different hosts and of same MLST type (Golubchik et al., 2013).

Prior studies tried to assess the number of SNPs accumulating over time, but mostly under selective conditions. Rouard et al. (2018) calculated that during selection for linezolid resistance an expected 17–93 mutations should accumulate per genome per year. A more global calculation using a significantly larger strain collection resulted in average number of less than 10 SNPs per genome per year (Harris et al., 2010). Ankrum and Hall (2017) came up with figures around 70 SNPs per year. Obviously, the discussion on epidemiological SNP cut off values defining identity (or not) or close relatedness between clinical isolates have not been finalized yet. On the basis of this study, we suggest a median SNP cut off 20 SNPs. Although in our study limited numbers of individuals are included, a high number of strains per individual were included to thoroughly study mutation frequency over time. Our suggested cut off can be used to identify *S. aureus* strains as identical or not in outbreak management.

Nasal colonization with strains carrying virulence determinants such as fibronectin (*fnb*) and collagen adhesions (*cna*) may represent risk for subsequent invasive infections in carriers (Peacock et al., 2002b; Nashev et al., 2004).

We acknowledge that our study is likely to be underpowered: limited numbers of individuals were able to take part in these long-lasting and logistically complicated studies. Still, the artificial inoculation model is a unique feature of this study. So far and except for our own work, very few studies have been done using artificial inoculation in humans (Cole et al., 2018). On the other hand, epidemiological studies usually take place in similar time frames as used here. The mutation frequency we observe here during weeks and months will be well aligned with those occurring during active outbreaks since these mostly also span weeks rather than months.

## Conclusion

Median core genome SNP counts and pairwise SNP divergence for all the strains studied here were always lower than 20 over periods up to 3 years of evolution in individual carriers. During artificial colonization, where median core genome SNP, and pairwise SNP distance scores were 2, there is no early stage selection of different genotypes. In addition, during stable long(er) term colonization (up to 3 years) the number of accumulating SNPs was low as well. We here suggest an epidemiological median cut off value of 20 SNPs as a marker of *S. aureus* strain identity during outbreaks of infection. Random pattern of pairwise SNP divergence between the strains isolated from single carrier suggested that the WGS of multiple colonies is necessary for outbreak infection analysis.

## Data Availability

The datasets for this manuscript are not publicly available because we are still in the process of submitting data on NCBI. Requests to access the datasets should be directed to manisha.goyal@biomerieux.com.

## Author Contributions

WvW, NV, and AvB conceived the study. MT conducted the microbiological experimentation for *S. aureus* strains. MG, FJ, MP, and CM carried out the whole genome sequencing studies. MG interpreted the sequence data and wrote the first version of the manuscript. All authors discussed the results and edited the manuscript.

## Conflict of Interest Statement

AvB, MP, CM, FJ, and MG were employees of bioMérieux, a company designing, developing, and marketing tests in the domain of infectious diseases. The company was not involved in the design of the current review and the opinions expressed are those of the authors and may be different from formal company opinions and policies. The remaining authors declare that the research was conducted in the absence of any commercial or financial relationships that could be construed as a potential conflict of interest.

## Footnotes

1 http://www.applied-maths.com/

2 https://pubmlst.org/saureus/

3 http://www.mgc.ac.cn/VFs/main.htm

4 http://www.genomicepidemiology.org/

5 https://github.com/tseemann/abricate
