# Supplementary material for: Genomic Evolution of Staphylococcus aureus During Artificial and Natural Colonization of the Human Nose
Source: Front Microbiol. 2019 Jul 5;10:1525. doi: 10.3389/fmicb.2019.01525 (PMC6624446; doi:10.3389/fmicb.2019.01525)
Supplement: Supplementary file 1 [file Table_1.DOCX]

Supplementary Material

# Supplementary Tables

**Supplementary Table 1.** Dataset used in this study along with the information of persistent *S. aureus* nasal carrier from which the strains were isolated and the date of their isolation. On each isolation date upto 3 colonies were picked and sequenced for each carrier.

| **Dataset** | | | | | | |
| --- | --- | --- | --- | --- | --- | --- |
| **Host ID** | **2007** | | **2008** | | **2010** | |
|  | *Isolate ID* | *Isolation date* | *Isolate ID* | *Isolation date* | *Isolate ID* | *Isolation date* |
| **A** | 1410027 | 13/03/2007 |  |  | 1410063 | 24/02/2010 |
|  | 1410028 | 13/03/2007 |  |  | 1410064 | 24/02/2010 |
|  | 1410029 | 13/03/2007 |  |  | 1410065 | 24/02/2010 |
|  | 1410030 | 05/06/2007 |  |  | 1410066 | 10/03/2010 |
|  | 1410031 | 05/06/2007 |  |  | 1410067 | 10/03/2010 |
|  | - | - |  |  | 1410068 | 10/03/2010 |
| **B** | 1410033 | 13/03/2007 |  |  | 1410069 | 02/03/2010 |
|  | 1410034 | 13/03/2007 |  |  | 1410070 | 02/03/2010 |
|  | 1410035 | 13/03/2007 |  |  | 1410071 | 02/03/2010 |
|  | 1410036 | 20/06/2007 |  |  | - | - |
|  | 1410037 | 20/06/2007 |  |  | 1410073 | 16/03/2010 |
|  | 1410038 | 20/06/2007 |  |  | 1410074 | 16/03/2010 |
| **C** | 1410039 | 26/03/2007 |  |  | 1410075 | 22/02/2010 |
|  | 1410040 | 26/03/2007 |  |  | 1410076 | 22/02/2010 |
|  | 1410041 | 26/03/2007 |  |  | 1410077 | 22/02/2010 |
|  | 1410042 | 04/06/2007 |  |  | 1410078 | 10/03/2010 |
|  | 1410043 | 04/06/2007 |  |  | 1410079 | 10/03/2010 |
|  | 1410044 | 04/06/2007 |  |  | 1410080 | 10/03/2010 |
| **D** | 1410045 | 13/03/2007 |  |  | 1410081 | 03/03/2010 |
|  | 1410046 | 13/03/2007 |  |  | 1410082 | 03/03/2010 |
|  | 1410047 | 13/03/2007 |  |  | 1410083 | 03/03/2010 |
|  | 1410048 | 15/06/2007 |  |  | 1410084 | 23/03/2010 |
|  | 1410049 | 15/06/2007 |  |  | 1410085 | 23/03/2010 |
|  | 1410050 | 15/06/2007 |  |  |  |  |
| **E** | 1410051 | 13/03/2007 |  |  |  |  |
|  | 1410052 | 13/03/2007 |  |  |  |  |
|  | 1410053 | 13/03/2007 |  |  |  |  |
|  | 1410054 | 10/04/2007 |  |  |  |  |
|  | 1410055 | 10/04/2007 |  |  |  |  |
|  | 1410056 | 10/04/2007 |  |  |  |  |
| **F** | 1410057 | 12/03/2007 |  |  |  |  |
|  | 1410058 | 12/03/2007 |  |  |  |  |
|  | 1410059 | 12/03/2007 |  |  |  |  |
|  | 1410060 | 04/06/2007 |  |  |  |  |
|  | 1410061 | 04/06/2007 |  |  |  |  |
|  | 1410062 | 04/06/2007 |  |  |  |  |
| **11A** |  |  | 1410099 | 01/01/2008 |  |  |
|  |  |  | 1410100 | 01/01/2008 |  |  |
|  |  |  | 1410101 | 01/01/2008 |  |  |
|  |  |  | 1410102 | 21/01/2008 |  |  |
| **12** |  |  | 1410106 | 01/01/2008 |  |  |
|  |  |  | 1410107 | 01/01/2008 |  |  |
|  |  |  | 1410108 | 01/01/2008 |  |  |
|  |  |  | 1410109 | 21/01/2008 |  |  |
|  |  |  | 1410110 | 21/01/2008 |  |  |
|  |  |  | 1410111 | 21/01/2008 |  |  |
| **16** |  |  | 1410112 | 01/01/2008 |  |  |
|  |  |  | 1410113 | 01/01/2008 |  |  |
|  |  |  | 1410114 | 01/01/2008 |  |  |
|  |  |  | 1410115 | 21/01/2008 |  |  |
|  |  |  | 1410116 | 21/01/2008 |  |  |
|  |  |  | 1410117 | 21/01/2008 |  |  |
| **18** |  |  | 1410118 | 01/01/2008 |  |  |
|  |  |  | 1410119 | 28/01/2008 |  |  |
|  |  |  | 1410120 | 28/01/2008 |  |  |
|  |  |  | 1410121 | 28/01/2008 |  |  |
| **19** |  |  | 1410122 | 01/01/2008 |  |  |
|  |  |  | 1410123 | 01/01/2008 |  |  |
|  |  |  | 1410124 | 01/01/2008 |  |  |
|  |  |  | 1410125 | 21/01/2008 |  |  |
| **20** |  |  | 1410126 | 01/01/2008 |  |  |
|  |  |  | 1410127 | 01/01/2008 |  |  |
|  |  |  | 1410128 | 01/01/2008 |  |  |
|  |  |  | 1410129 | 06/01/2008 |  |  |

**Supplementary Table 2.** Whole genome sequencing quality assessment of complete dataset (2007, 2008 and 2010).

| **_SEQ ID_** | **_Contigs_** | **_Scaffolds_** | **_Genome Size_** | **_Longest Scaffold_** | **_N50_** | **_Raw reads_** | **_EC Reads_** | **_% reads passing EC_** | **_Raw nt_** | **_EC nt_** | **_% nt passing EC_** | **_Raw cov_** | **_Median cov_** | **_10th percentile cov_** | **_bases >= Q40_** | **_assembler version_** |
| --- | --- | --- | --- | --- | --- | --- | --- | --- | --- | --- | --- | --- | --- | --- | --- | --- |
| **_1410027_** | _73_ | _52_ | _2859794_ | _408220_ | _125209_ | _1933334_ | _1920040_ | _99.31_ | _290000100_ | _2.62E+08_ | _90.33_ | _91.6_ | _85_ | _32_ | _2854454_ | _A5-miseq 20140604_ |
| **_1410028_** | _68_ | _68_ | _2854262_ | _351421_ | _83311_ | _1933334_ | _1904270_ | _98.5_ | _290000100_ | _2.24E+08_ | _77.36_ | _78.6_ | _68_ | _29_ | _2847967_ | _A5-miseq 20140604_ |
| **_1410029_** | _59_ | _56_ | _2857862_ | _406166_ | _134664_ | _1933334_ | _1905898_ | _98.58_ | _290000100_ | _2.29E+08_ | _78.93_ | _80.1_ | _77_ | _42_ | _2856116_ | _A5-miseq 20140604_ |
| **_1410030_** | _71_ | _61_ | _2859900_ | _323908_ | _100453_ | _1933334_ | _1900960_ | _98.33_ | _290000100_ | _2.31E+08_ | _79.65_ | _80.77_ | _72_ | _33_ | _2856178_ | _A5-miseq 20140604_ |
| **_1410031_** | _71_ | _72_ | _2859717_ | _304300_ | _84101_ | _1933334_ | _1899989_ | _98.28_ | _290000100_ | _2.3E+08_ | _79.22_ | _80.34_ | _71_ | _31_ | _2855699_ | _A5-miseq 20140604_ |
| **_1410033_** | _57_ | _39_ | _2892577_ | _403413_ | _137825_ | _1933334_ | _1911291_ | _98.86_ | _290000100_ | _2.44E+08_ | _83.99_ | _84.21_ | _77_ | _36_ | _2890924_ | _A5-miseq 20140604_ |
| **_1410034_** | _61_ | _46_ | _2893198_ | _437294_ | _149696_ | _1933334_ | _1909974_ | _98.79_ | _290000100_ | _2.39E+08_ | _82.37_ | _82.57_ | _79_ | _43_ | _2891995_ | _A5-miseq 20140604_ |
| **_1410035_** | _62_ | _48_ | _2893457_ | _536467_ | _170686_ | _1933334_ | _1906313_ | _98.6_ | _290000100_ | _2.35E+08_ | _81.17_ | _81.35_ | _72_ | _35_ | _2891385_ | _A5-miseq 20140604_ |
| **_1410036_** | _65_ | _36_ | _2894807_ | _412544_ | _201545_ | _1933334_ | _1913625_ | _98.98_ | _290000100_ | _2.57E+08_ | _88.45_ | _88.61_ | _86_ | _42_ | _2893268_ | _A5-miseq 20140604_ |
| **_1410037_** | _66_ | _42_ | _2893772_ | _383228_ | _175083_ | _1933334_ | _1907081_ | _98.64_ | _290000100_ | _2.45E+08_ | _84.63_ | _84.81_ | _78_ | _39_ | _2892277_ | _A5-miseq 20140604_ |
| **_1410038_** | _110_ | _106_ | _2910024_ | _207327_ | _71183_ | _1933334_ | _1904243_ | _98.5_ | _290000100_ | _2.27E+08_ | _78.43_ | _78.16_ | _67_ | _26_ | _2893344_ | _A5-miseq 20140604_ |
| **_1410039_** | _69_ | _69_ | _2740888_ | _324459_ | _127426_ | _1933334_ | _1901850_ | _98.37_ | _290000100_ | _2.26E+08_ | _78.09_ | _82.62_ | _71_ | _28_ | _2733501_ | _A5-miseq 20140604_ |
| **_1410040_** | _73_ | _73_ | _2740823_ | _271545_ | _117037_ | _1933334_ | _1902873_ | _98.42_ | _290000100_ | _2.23E+08_ | _77.05_ | _81.53_ | _70_ | _30_ | _2735346_ | _A5-miseq 20140604_ |
| **_1410041_** | _72_ | _72_ | _2740899_ | _195838_ | _99059_ | _1933334_ | _1903983_ | _98.48_ | _290000100_ | _2.2E+08_ | _75.83_ | _80.23_ | _68_ | _26_ | _2732517_ | _A5-miseq 20140604_ |
| **_1410042_** | _55_ | _55_ | _2737358_ | _367688_ | _159046_ | _1933334_ | _1898175_ | _98.18_ | _290000100_ | _2.31E+08_ | _79.54_ | _84.26_ | _78_ | _37_ | _2735351_ | _A5-miseq 20140604_ |
| **_1410043_** | _61_ | _60_ | _2740123_ | _258332_ | _128525_ | _1933334_ | _1897785_ | _98.16_ | _290000100_ | _2.27E+08_ | _78.33_ | _82.9_ | _73_ | _32_ | _2736727_ | _A5-miseq 20140604_ |
| **_1410044_** | _54_ | _18_ | _2740007_ | _1367212_ | _444227_ | _1933334_ | _1894815_ | _98.01_ | _290000100_ | _2.62E+08_ | _90.51_ | _95.8_ | _95_ | _43_ | _2738779_ | _A5-miseq 20140604_ |
| **_1410045_** | _50_ | _46_ | _2753752_ | _464338_ | _229055_ | _1933334_ | _1906151_ | _98.59_ | _290000100_ | _2.35E+08_ | _81.01_ | _85.31_ | _77_ | _37_ | _2751376_ | _A5-miseq 20140604_ |
| **_1410046_** | _57_ | _53_ | _2755612_ | _309594_ | _156112_ | _1933334_ | _1906344_ | _98.6_ | _290000100_ | _2.3E+08_ | _79.46_ | _83.62_ | _81_ | _44_ | _2754320_ | _A5-miseq 20140604_ |
| **_1410047_** | _56_ | _56_ | _2754465_ | _668832_ | _155081_ | _1933334_ | _1901326_ | _98.34_ | _290000100_ | _2.28E+08_ | _78.49_ | _82.63_ | _72_ | _35_ | _2752083_ | _A5-miseq 20140604_ |
| **_1410048_** | _51_ | _33_ | _2754424_ | _837835_ | _304730_ | _1933334_ | _1911359_ | _98.86_ | _290000100_ | _2.51E+08_ | _86.66_ | _91.24_ | _90_ | _43_ | _2752910_ | _A5-miseq 20140604_ |
| **_1410049_** | _50_ | _28_ | _2754558_ | _840688_ | _610740_ | _1933334_ | _1905322_ | _98.55_ | _290000100_ | _2.44E+08_ | _84.17_ | _88.61_ | _87_ | _43_ | _2753428_ | _A5-miseq 20140604_ |
| **_1410050_** | _63_ | _63_ | _2756327_ | _299870_ | _148468_ | _1933334_ | _1904061_ | _98.49_ | _290000100_ | _2.28E+08_ | _78.52_ | _82.61_ | _73_ | _32_ | _2751357_ | _A5-miseq 20140604_ |
| **_1410051_** | _74_ | _73_ | _2889940_ | _339254_ | _111343_ | _1933334_ | _1907684_ | _98.67_ | _290000100_ | _2.32E+08_ | _79.98_ | _80.26_ | _70_ | _31_ | _2886298_ | _A5-miseq 20140604_ |
| **_1410052_** | _82_ | _82_ | _2847234_ | _181577_ | _81820_ | _1933334_ | _1904662_ | _98.52_ | _290000100_ | _2.24E+08_ | _77.09_ | _78.51_ | _67_ | _30_ | _2842191_ | _A5-miseq 20140604_ |
| **_1410053_** | _83_ | _83_ | _2846249_ | _181577_ | _67698_ | _1933334_ | _1902853_ | _98.42_ | _290000100_ | _2.18E+08_ | _75.17_ | _76.59_ | _64_ | _28_ | _2839957_ | _A5-miseq 20140604_ |
| **_1410054_** | _72_ | _49_ | _2889655_ | _394320_ | _136643_ | _1933334_ | _1898447_ | _98.2_ | _290000100_ | _2.36E+08_ | _81.54_ | _81.83_ | _76_ | _42_ | _2887663_ | _A5-miseq 20140604_ |
| **_1410055_** | _66_ | _60_ | _2853850_ | _390046_ | _111343_ | _1933334_ | _1899186_ | _98.23_ | _290000100_ | _2.31E+08_ | _79.69_ | _80.98_ | _72_ | _35_ | _2851292_ | _A5-miseq 20140604_ |
| **_1410056_** | _73_ | _35_ | _2881424_ | _538542_ | _460591_ | _1933334_ | _1882895_ | _97.39_ | _290000100_ | _2.66E+08_ | _91.69_ | _92.28_ | _91_ | _46_ | _2879216_ | _A5-miseq 20140604_ |
| **_1410057_** | _42_ | _42_ | _2710715_ | _411317_ | _200829_ | _1933334_ | _1898392_ | _98.19_ | _290000100_ | _2.2E+08_ | _75.96_ | _81.27_ | _69_ | _37_ | _2709096_ | _A5-miseq 20140604_ |
| **_1410058_** | _39_ | _38_ | _2709876_ | _470980_ | _191330_ | _1933334_ | _1900338_ | _98.29_ | _290000100_ | _2.26E+08_ | _77.8_ | _83.25_ | _70_ | _35_ | _2708561_ | _A5-miseq 20140604_ |
| **_1410059_** | _36_ | _30_ | _2709226_ | _532201_ | _200843_ | _1933334_ | _1903669_ | _98.47_ | _290000100_ | _2.33E+08_ | _80.33_ | _85.99_ | _75_ | _36_ | _2707717_ | _A5-miseq 20140604_ |
| **_1410060_** | _315_ | _258_ | _2922365_ | _548010_ | _107697_ | _1933334_ | _1907791_ | _98.68_ | _290000100_ | _2.34E+08_ | _80.59_ | _79.97_ | _72_ | _22_ | _2843675_ | _A5-miseq 20140604_ |
| **_1410061_** | _40_ | _40_ | _2710010_ | _470980_ | _191330_ | _1933334_ | _1899782_ | _98.26_ | _290000100_ | _2.26E+08_ | _78.08_ | _83.55_ | _72_ | _35_ | _2707941_ | _A5-miseq 20140604_ |
| **_1410062_** | _58_ | _58_ | _2711785_ | _200843_ | _85574_ | _1933334_ | _1902170_ | _98.39_ | _290000100_ | _2.21E+08_ | _76.06_ | _81.33_ | _70_ | _30_ | _2706247_ | _A5-miseq 20140604_ |
| **_1410063_** | _70_ | _70_ | _2814063_ | _351616_ | _104493_ | _1933334_ | _1907427_ | _98.66_ | _290000100_ | _2.28E+08_ | _78.51_ | _80.91_ | _69_ | _29_ | _2807701_ | _A5-miseq 20140604_ |
| **_1410064_** | _68_ | _46_ | _2814465_ | _394324_ | _150972_ | _1933334_ | _1912153_ | _98.9_ | _290000100_ | _2.4E+08_ | _82.75_ | _85.27_ | _76_ | _36_ | _2811192_ | _A5-miseq 20140604_ |
| **_1410065_** | _65_ | _43_ | _2813459_ | _548014_ | _151301_ | _1933334_ | _1917703_ | _99.19_ | _290000100_ | _2.53E+08_ | _87.19_ | _89.87_ | _83_ | _38_ | _2810695_ | _A5-miseq 20140604_ |
| **_1410066_** | _60_ | _57_ | _2813450_ | _409520_ | _116313_ | _1933334_ | _1894411_ | _97.99_ | _290000100_ | _2.31E+08_ | _79.51_ | _81.96_ | _72_ | _37_ | _2811345_ | _A5-miseq 20140604_ |
| **_1410067_** | _67_ | _64_ | _2815592_ | _307750_ | _111460_ | _1933334_ | _1892353_ | _97.88_ | _290000100_ | _2.27E+08_ | _78.15_ | _80.5_ | _72_ | _38_ | _2813402_ | _A5-miseq 20140604_ |
| **_1410068_** | _66_ | _46_ | _2814675_ | _461304_ | _153028_ | _1933334_ | _1891534_ | _97.84_ | _290000100_ | _2.42E+08_ | _83.58_ | _86.12_ | _82_ | _39_ | _2811765_ | _A5-miseq 20140604_ |
| **_1410069_** | _64_ | _64_ | _2782717_ | _258729_ | _111581_ | _1933334_ | _1896933_ | _98.12_ | _290000100_ | _2.22E+08_ | _76.41_ | _79.63_ | _77_ | _38_ | _2781044_ | _A5-miseq 20140604_ |
| **_1410070_** | _62_ | _62_ | _2781905_ | _279310_ | _115193_ | _1933334_ | _1899907_ | _98.27_ | _290000100_ | _2.25E+08_ | _77.51_ | _80.8_ | _77_ | _40_ | _2780477_ | _A5-miseq 20140604_ |
| **_1410071_** | _79_ | _79_ | _2784671_ | _171843_ | _108349_ | _1933334_ | _1896509_ | _98.1_ | _290000100_ | _2.19E+08_ | _75.45_ | _78.57_ | _67_ | _30_ | _2779024_ | _A5-miseq 20140604_ |
| **_1410073_** | _71_ | _71_ | _2783790_ | _244407_ | _105411_ | _1933334_ | _1897815_ | _98.16_ | _290000100_ | _2.25E+08_ | _77.7_ | _80.95_ | _71_ | _33_ | _2780175_ | _A5-miseq 20140604_ |
| **_1410074_** | _100_ | _100_ | _2784905_ | _157561_ | _73330_ | _1933334_ | _1905281_ | _98.55_ | _290000100_ | _2.24E+08_ | _77.16_ | _80.34_ | _70_ | _27_ | _2773231_ | _A5-miseq 20140604_ |
| **_1410075_** | _60_ | _23_ | _2741877_ | _640829_ | _263418_ | _1933334_ | _1918829_ | _99.25_ | _290000100_ | _2.62E+08_ | _90.47_ | _95.69_ | _93_ | _42_ | _2739588_ | _A5-miseq 20140604_ |
| **_1410076_** | _58_ | _55_ | _2740157_ | _258332_ | _128524_ | _1933334_ | _1911053_ | _98.85_ | _290000100_ | _2.34E+08_ | _80.61_ | _85.32_ | _77_ | _35_ | _2737111_ | _A5-miseq 20140604_ |
| **_1410077_** | _62_ | _62_ | _2738785_ | _283213_ | _104463_ | _1933334_ | _1906276_ | _98.6_ | _290000100_ | _2.27E+08_ | _78.23_ | _82.83_ | _72_ | _31_ | _2734542_ | _A5-miseq 20140604_ |
| **_1410078_** | _56_ | _50_ | _2740963_ | _324582_ | _137554_ | _1933334_ | _1896432_ | _98.09_ | _290000100_ | _2.29E+08_ | _79.09_ | _83.68_ | _81_ | _47_ | _2739930_ | _A5-miseq 20140604_ |
| **_1410079_** | _63_ | _57_ | _2741747_ | _483675_ | _137554_ | _1933334_ | _1896384_ | _98.09_ | _290000100_ | _2.3E+08_ | _79.25_ | _83.83_ | _76_ | _40_ | _2740056_ | _A5-miseq 20140604_ |
| **_1410080_** | _53_ | _20_ | _2740015_ | _761948_ | _498068_ | _1933334_ | _1891110_ | _97.82_ | _290000100_ | _2.46E+08_ | _84.94_ | _89.9_ | _90_ | _53_ | _2739315_ | _A5-miseq 20140604_ |
| **_1410081_** | _63_ | _49_ | _2720565_ | _507031_ | _160404_ | _1933334_ | _1899390_ | _98.24_ | _290000100_ | _2.25E+08_ | _77.67_ | _82.8_ | _80_ | _45_ | _2719131_ | _A5-miseq 20140604_ |
| **_1410082_** | _58_ | _44_ | _2719039_ | _511450_ | _147264_ | _1933334_ | _1898986_ | _98.22_ | _290000100_ | _2.25E+08_ | _77.7_ | _82.87_ | _80_ | _45_ | _2718213_ | _A5-miseq 20140604_ |
| **_1410084_** | _65_ | _38_ | _2721154_ | _503333_ | _174086_ | _1933334_ | _1909078_ | _98.75_ | _290000100_ | _2.39E+08_ | _82.33_ | _87.74_ | _85_ | _51_ | _2720582_ | _A5-miseq 20140604_ |
| **_1410085_** | _65_ | _28_ | _2719503_ | _503667_ | _252044_ | _1933334_ | _1914789_ | _99.04_ | _290000100_ | _2.51E+08_ | _86.72_ | _92.48_ | _87_ | _32_ | _2716037_ | _A5-miseq 20140604_ |
| **_1410099_** | _53_ | _53_ | _2671406_ | _310078_ | _159510_ | _1933334_ | _1904862_ | _98.53_ | _290000100_ | _2.26E+08_ | _77.78_ | _84.43_ | _74_ | _30_ | _2666918_ | _A5-miseq 20140604_ |
| **_1410100_** | _43_ | _43_ | _2671235_ | _833179_ | _237772_ | _1933334_ | _1905607_ | _98.57_ | _290000100_ | _2.32E+08_ | _79.97_ | _86.82_ | _84_ | _48_ | _2670652_ | _A5-miseq 20140604_ |
| **_1410101_** | _56_ | _37_ | _2672209_ | _384098_ | _169646_ | _1933334_ | _1917267_ | _99.17_ | _290000100_ | _2.57E+08_ | _88.62_ | _96.17_ | _90_ | _33_ | _2667569_ | _A5-miseq 20140604_ |
| **_1410102_** | _59_ | _59_ | _2674419_ | _452400_ | _148463_ | _1933334_ | _1897303_ | _98.14_ | _290000100_ | _2.25E+08_ | _77.67_ | _84.22_ | _76_ | _34_ | _2670963_ | _A5-miseq 20140604_ |
| **_1410106_** | _58_ | _58_ | _2671978_ | _390111_ | _112844_ | _1933334_ | _1900569_ | _98.31_ | _290000100_ | _2.24E+08_ | _77.07_ | _83.65_ | _74_ | _29_ | _2666554_ | _A5-miseq 20140604_ |
| **_1410107_** | _66_ | _66_ | _2673043_ | _299497_ | _106833_ | _1933334_ | _1900277_ | _98.29_ | _290000100_ | _2.19E+08_ | _75.38_ | _81.78_ | _70_ | _27_ | _2666761_ | _A5-miseq 20140604_ |
| **_1410108_** | _73_ | _73_ | _2673123_ | _285019_ | _100219_ | _1933334_ | _1898874_ | _98.22_ | _290000100_ | _2.18E+08_ | _75.09_ | _81.46_ | _70_ | _27_ | _2666153_ | _A5-miseq 20140604_ |
| **_1410109_** | _79_ | _79_ | _2672637_ | _197504_ | _83216_ | _1933334_ | _1896667_ | _98.1_ | _290000100_ | _2.15E+08_ | _74.29_ | _80.61_ | _70_ | _27_ | _2663575_ | _A5-miseq 20140604_ |
| **_1410110_** | _71_ | _71_ | _2673996_ | _286671_ | _109210_ | _1933334_ | _1900180_ | _98.29_ | _290000100_ | _2.15E+08_ | _74.3_ | _80.58_ | _70_ | _26_ | _2667672_ | _A5-miseq 20140604_ |
| **_1410111_** | _51_ | _51_ | _2672386_ | _355612_ | _148457_ | _1933334_ | _1907165_ | _98.65_ | _290000100_ | _2.28E+08_ | _78.68_ | _85.38_ | _75_ | _30_ | _2667735_ | _A5-miseq 20140604_ |
| **_1410112_** | _44_ | _44_ | _2671969_ | _452294_ | _159515_ | _1933334_ | _1907091_ | _98.64_ | _290000100_ | _2.31E+08_ | _79.62_ | _86.41_ | _77_ | _41_ | _2670433_ | _A5-miseq 20140604_ |
| **_1410113_** | _51_ | _19_ | _2673562_ | _1161201_ | _673680_ | _1933334_ | _1919688_ | _99.29_ | _290000100_ | _2.69E+08_ | _92.6_ | _100.44_ | _96_ | _34_ | _2670331_ | _A5-miseq 20140604_ |
| **_1410114_** | _53_ | _38_ | _2673442_ | _308308_ | _148463_ | _1933334_ | _1912704_ | _98.93_ | _290000100_ | _2.48E+08_ | _85.37_ | _92.6_ | _85_ | _36_ | _2670003_ | _A5-miseq 20140604_ |
| **_1410115_** | _52_ | _52_ | _2672239_ | _621715_ | _125830_ | _1933334_ | _1896208_ | _98.08_ | _290000100_ | _2.21E+08_ | _76.18_ | _82.67_ | _72_ | _31_ | _2668901_ | _A5-miseq 20140604_ |
| **_1410116_** | _45_ | _20_ | _2672715_ | _850556_ | _543596_ | _1933334_ | _1894523_ | _97.99_ | _290000100_ | _2.48E+08_ | _85.68_ | _92.97_ | _92_ | _45_ | _2671950_ | _A5-miseq 20140604_ |
| **_1410117_** | _56_ | _56_ | _2673807_ | _310481_ | _148463_ | _1933334_ | _1905611_ | _98.57_ | _290000100_ | _2.32E+08_ | _79.98_ | _86.75_ | _78_ | _33_ | _2670539_ | _A5-miseq 20140604_ |
| **_1410118_** | _50_ | _50_ | _2673055_ | _620030_ | _148457_ | _1933334_ | _1907074_ | _98.64_ | _290000100_ | _2.32E+08_ | _79.83_ | _86.61_ | _84_ | _41_ | _2672060_ | _A5-miseq 20140604_ |
| **_1410119_** | _45_ | _34_ | _2672298_ | _578139_ | _239576_ | _1933334_ | _1911941_ | _98.89_ | _290000100_ | _2.46E+08_ | _84.92_ | _92.16_ | _86_ | _36_ | _2669825_ | _A5-miseq 20140604_ |
| **_1410120_** | _52_ | _52_ | _2672393_ | _450883_ | _145538_ | _1933334_ | _1899838_ | _98.27_ | _290000100_ | _2.24E+08_ | _77.11_ | _83.67_ | _74_ | _33_ | _2669192_ | _A5-miseq 20140604_ |
| **_1410121_** | _54_ | _54_ | _2671075_ | _161331_ | _113916_ | _1933334_ | _1901116_ | _98.33_ | _290000100_ | _2.27E+08_ | _78.22_ | _84.92_ | _75_ | _31_ | _2666528_ | _A5-miseq 20140604_ |
| **_1410122_** | _76_ | _83_ | _2673349_ | _165701_ | _66437_ | _1933334_ | _1902868_ | _98.42_ | _290000100_ | _2.19E+08_ | _75.48_ | _81.88_ | _72_ | _28_ | _2666031_ | _A5-miseq 20140604_ |
| **_1410123_** | _54_ | _54_ | _2670728_ | _235672_ | _109614_ | _1933334_ | _1907675_ | _98.67_ | _290000100_ | _2.23E+08_ | _76.97_ | _83.58_ | _72_ | _29_ | _2666216_ | _A5-miseq 20140604_ |
| **_1410124_** | _53_ | _53_ | _2672221_ | _531956_ | _237772_ | _1933334_ | _1904150_ | _98.49_ | _290000100_ | _2.27E+08_ | _78.13_ | _84.79_ | _75_ | _39_ | _2670401_ | _A5-miseq 20140604_ |
| **_1410125_** | _50_ | _31_ | _2672890_ | _582009_ | _249650_ | _1933334_ | _1909828_ | _98.78_ | _290000100_ | _2.45E+08_ | _84.57_ | _91.76_ | _84_ | _33_ | _2669783_ | _A5-miseq 20140604_ |
| **_1410126_** | _50_ | _43_ | _2673031_ | _545850_ | _159515_ | _1933334_ | _1912056_ | _98.9_ | _290000100_ | _2.42E+08_ | _83.32_ | _90.39_ | _86_ | _36_ | _2671014_ | _A5-miseq 20140604_ |
| **_1410127_** | _61_ | _61_ | _2673069_ | _304168_ | _116823_ | _1933334_ | _1906820_ | _98.63_ | _290000100_ | _2.3E+08_ | _79.38_ | _86.12_ | _75_ | _29_ | _2667483_ | _A5-miseq 20140604_ |
| **_1410128_** | _53_ | _53_ | _2672993_ | _310079_ | _155775_ | _1933334_ | _1905988_ | _98.59_ | _290000100_ | _2.31E+08_ | _79.71_ | _86.47_ | _76_ | _30_ | _2668876_ | _A5-miseq 20140604_ |
| **_1410129_** | _52_ | _52_ | _2671590_ | _619955_ | _145412_ | _1933334_ | _1903793_ | _98.47_ | _290000100_ | _2.26E+08_ | _77.85_ | _84.5_ | _73_ | _30_ | _2667589_ | _A5-miseq 20140604_ |

**Supplementary Table 3.** Resistance and virulence genes identified in *S. aureus* isolates of entire dataset.

| **Isolate ID** | **Isolation date** | **Host ID** | **Virulence Genes** | **Resistance Genes** |
| --- | --- | --- | --- | --- |
| **1410027** | 13/03/2007 | A | sea, hla, hlb, hld, hlgB, clfA, clfB, fnbA, icaA, sdrC, sdrE, tsst-1 | blaZ |
| **1410028** | 13/03/2007 | A | sea, hla, hlb, hld, hlgB, clfA, clfB, fnbA, icaA, sdrC, sdrE, tsst-1 | blaZ |
| **1410029** | 13/03/2007 | A | sea, hla, hlb, hld, hlgB, clfA, clfB, fnbA, icaA, sdrC, sdrE, tsst-1 | blaZ |
| **1410030** | 05/06/2007 | A | sea, hla, hlb, hld, hlgB, clfA, clfB, fnbA, icaA, sdrC, sdrE, tsst-1 | blaZ |
| **1410031** | 05/06/2007 | A | sea, hla, hlb, hld, hlgB, clfA, clfB, fnbA, icaA, sdrC, sdrE, tsst-1 | blaZ |
| **1410033** | 13/03/2007 | B | hla, hlb, hld, hlgB, clfA, clfB, fnbA, icaA, sdrC, sdrE, tsst-1, sdrD | blaZ, erm(C), tet(M) |
| **1410034** | 13/03/2007 | B | hla, hlb, hld, hlgB, clfA, clfB, fnbA, icaA, sdrC, sdrE, tsst-1, sdrD | blaZ, erm(C), tet(M) |
| **1410035** | 13/03/2007 | B | hla, hlb, hld, hlgB, clfA, clfB, fnbA, icaA, sdrC, sdrE, tsst-1, sdrD | blaZ, erm(C), tet(M) |
| **1410036** | 20/06/2007 | B | hla, hlb, hld, hlgB, clfA, clfB, fnbA, icaA, sdrC, sdrE, tsst-1, sdrD | blaZ, erm(C), tet(M) |
| **1410037** | 20/06/2007 | B | hla, hlb, hld, hlgB, clfA, clfB, fnbA, icaA, sdrC, sdrE, tsst-1, sdrD | blaZ, erm(C), tet(M) |
| **1410038** | 20/06/2007 | B | hla, hlb, hld, hlgB, clfA, clfB, fnbA, icaA, sdrC, sdrE, tsst-1, sdrD | blaZ, erm(C), tet(M) |
| **1410039** | 26/03/2007 | C | hla, hlb, hld, hlgB, clfA, clfB, fnbA, icaA, sdrC, fnbB | blaZ |
| **1410040** | 26/03/2007 | C | hla, hlb, hld, hlgB, clfA, clfB, fnbA, icaA, sdrC, sdrE, fnbB | blaZ |
| **1410041** | 26/03/2007 | C | hla, hlb, hld, hlgB, clfA, clfB, fnbA, icaA, sdrC, fnbB | blaZ |
| **1410042** | 04/06/2007 | C | hla, hlb, hld, hlgB, clfA, clfB, fnbA, icaA, sdrC, fnbB | blaZ |
| **1410043** | 04/06/2007 | C | hla, hlb, hld, hlgB, clfA, clfB, fnbA, icaA, sdrC, fnbB | blaZ |
| **1410044** | 04/06/2007 | C | hla, hlb, hld, hlgB, clfA, fnbA, icaA, sdrC, fnbB | blaZ |
| **1410045** | 13/03/2007 | D | hla, hlb, hld, hlgB, clfA, clfB, fnbA, icaA, sdrC, fnbB, sdrE, sdrD | None |
| **1410046** | 13/03/2007 | D | hla, hlb, hld, hlgB, clfA, clfB, fnbA, icaA, sdrC, fnbB, sdrE, sdrD | None |
| **1410047** | 13/03/2007 | D | hla, hlb, hld, hlgB, clfA, clfB, fnbA, icaA, sdrC, fnbB, sdrE, sdrD | None |
| **1410048** | 15/06/2007 | D | hla, hlb, hld, hlgB, clfA, clfB, fnbA, icaA, sdrC, fnbB, sdrE, sdrD | None |
| **1410049** | 15/06/2007 | D | hla, hlb, hld, hlgB, clfA, clfB, fnbA, icaA, sdrC, fnbB, sdrE, sdrD | None |
| **1410050** | 15/06/2007 | D | hla, hlb, hld, hlgB, clfA, clfB, fnbA, icaA, sdrC, fnbB, sdrE, sdrD | None |
| **1410051** | 13/03/2007 | E | hla, hlb, hld, hlgB, clfA, clfB, fnbA, icaA, sdrC, sdrE, sdrD, tsst-1 | blaZ |
| **1410052** | 13/03/2007 | E | hla, hlb, hld, hlgB, clfA, clfB, fnbA, icaA, sdrC, sdrE, sdrD, tsst-1 | blaZ |
| **1410053** | 13/03/2007 | E | hla, hlb, hld, hlgB, clfA, clfB, fnbA, icaA, sdrC, sdrE, sdrD, tsst-1 | blaZ |
| **1410054** | 10/04/2007 | E | hla, hlb, hld, hlgB, clfA, clfB, fnbA, icaA, sdrC, sdrE, sdrD, tsst-1, cna | blaZ |
| **1410055** | 10/04/2007 | E | hla, hlb, hld, hlgB, clfA, clfB, fnbA, icaA, sdrC, sdrE, cna | blaZ |
| **1410056** | 10/04/2007 | E | hla, hlb, hld, hlgB, clfA, clfB, fnbA, icaA, sdrC, sdrE, sdrD, tsst-1 | blaZ |
| **1410057** | 12/03/2007 | F | hla, hlb, hld, hlgB, clfA, clfB, fnbA, icaA, sdrC, fnbB, sdrE, sdrD, tsst-1 | blaZ, tet(M) |
| **1410058** | 12/03/2007 | F | hla, hlb, hld, hlgB, clfA, clfB, fnbA, icaA, sdrC, fnbB, sdrE, sdrD, tsst-1 | blaZ, tet(M) |
| **1410059** | 12/03/2007 | F | hla, hlb, hld, hlgB, clfA, clfB, fnbA, icaA, sdrC, fnbB, sdrE, sdrD, tsst-1 | blaZ, tet(M) |
| **1410060** | 04/06/2007 | F | hla, hlb, hld, hlgB, clfA, clfB, icaA, sdrC, fnbB, sdrD, tsst-1 | tet(M) |
| **1410061** | 04/06/2007 | F | hla, hlb, hld, hlgB, clfA, clfB, fnbA, icaA, sdrC, fnbB, sdrE, sdrD, tsst-1 | blaZ, tet(M) |
| **1410062** | 04/06/2007 | F | hla, hlb, hld, hlgB, clfA, clfB, fnbA, icaA, sdrC, fnbB, sdrE, sdrD, tsst-1 | blaZ, tet(M) |
| **1410063** | 24/02/2010 | A | hla, hlb, hld, hlgB, clfA, clfB, icaA, sdrC, fnbA, sdrE, tsst-1 | blaZ |
| **1410064** | 24/02/2010 | A | hla, hlb, hld, hlgB, clfA, clfB, icaA, sdrC, fnbA, sdrE, tsst-1 | blaZ |
| **1410065** | 24/02/2010 | A | hla, hlb, hld, hlgB, clfA, clfB, icaA, sdrC, fnbA, tsst-1 | blaZ |
| **1410066** | 10/03/2010 | A | hla, hlb, hld, hlgB, clfA, clfB, icaA, sdrC, fnbA, sdrE, tsst-1 | blaZ |
| **1410067** | 10/03/2010 | A | hla, hlb, hld, hlgB, clfA, clfB, icaA, sdrC, fnbA, sdrE, tsst-1 | blaZ |
| **1410068** | 10/03/2010 | A | hla, hlb, hld, hlgB, clfA, clfB, icaA, sdrC, fnbA, sdrE, tsst-1 | blaZ |
| **1410069** | 02/03/2010 | B | hla, hlb, hld, hlgB, clfA, clfB, icaA, sdrC, sea, sdrD, sdrE | blaZ |
| **1410070** | 02/03/2010 | B | hla, hlb, hld, hlgB, clfA, clfB, icaA, sdrC, sea, sdrD, sdrE | blaZ |
| **1410071** | 02/03/2010 | B | hla, hlb, hld, hlgB, clfA, clfB, icaA, sdrC, sea, sdrD, sdrE | blaZ |
| **1410073** | 16/03/2010 | B | hla, hlb, hld, hlgB, clfA, clfB, icaA, sdrC, sea, sdrD, sdrE | blaZ |
| **1410074** | 16/03/2010 | B | hla, hlb, hld, hlgB, clfA, clfB, icaA, sdrC, sea, sdrD, sdrE | blaZ |
| **1410075** | 22/02/2010 | C | hla, hlb, hld, hlgB, clfA, clfB, icaA, sdrC, fnbA, fnbB | blaZ |
| **1410076** | 22/02/2010 | C | hla, hlb, hld, hlgB, clfA, clfB, icaA, sdrC, fnbA, fnbB | blaZ |
| **1410077** | 22/02/2010 | C | hla, hlb, hld, hlgB, clfA, clfB, icaA, sdrC, fnbA, fnbB | blaZ |
| **1410078** | 10/03/2010 | C | hla, hlb, hld, hlgB, clfA, clfB, icaA, sdrC, fnbA, fnbB | blaZ |
| **1410079** | 10/03/2010 | C | hla, hlb, hld, hlgB, clfA, clfB, icaA, sdrC, fnbA, fnbB | blaZ |
| **1410080** | 10/03/2010 | C | hla, hlb, hld, hlgB, clfA, clfB, icaA, sdrC, fnbA, fnbB | blaZ |
| **1410081** | 03/03/2010 | D | hla, hlb, hld, hlgB, clfA, clfB, icaA, sdrC, fnbA, fnbB,sdrD, sdrE | None |
| **1410082** | 03/03/2010 | D | hla, hlb, hld, hlgB, clfA, clfB, icaA, sdrC, fnbA, fnbB,sdrD, sdrE | None |
| **1410083** | 03/03/2010 | D | hla, hlb, hld, hlgB, clfA, icaA, sdrC, fnbA, fnbB,sdrD, sdrE | None |
| **1410084** | 23/03/2010 | D | hla, hlb, hld, hlgB, clfA, clfB, icaA, sdrC, fnbA, fnbB,sdrD, sdrE | None |
| **1410085** | 23/03/2010 | D | hla, hlb, hld, hlgB, clfA, clfB, icaA, sdrC, fnbA, fnbB,sdrD, sdrE | None |
| **1410099** | 01/01/2008 | 11A | hla, hlb, hld, hlgB, clfA, clfB, fnbA, fnbB icaA, sdrC, sdrD | None |
| **1410100** | 01/01/2008 | 11A | hla, hlb, hld, hlgB, clfA, clfB, fnbA, fnbB icaA, sdrC, sdrD | None |
| **1410101** | 01/01/2008 | 11A | hla, hlb, hld, hlgB, clfA, clfB, fnbA, fnbB icaA, sdrC, sdrD | None |
| **1410102** | 21/01/2008 | 11A | hla, hlb, hld, hlgB, clfA, clfB, fnbA, fnbB icaA, sdrC, sdrD | None |
| **1410106** | 01/01/2008 | 12 | hla, hlb, hld, hlgB, clfA, clfB, fnbA, fnbB icaA, sdrC, sdrD | None |
| **1410107** | 01/01/2008 | 12 | hla, hlb, hld, hlgB, clfA, clfB, fnbA, fnbB icaA, sdrC, sdrD | None |
| **1410108** | 01/01/2008 | 12 | hla, hlb, hld, hlgB, clfA, clfB, fnbA, fnbB icaA, sdrC, sdrD | None |
| **1410109** | 21/01/2008 | 12 | hla, hlb, hld, hlgB, clfA, clfB, fnbA, fnbB icaA, sdrC, sdrD | None |
| **1410110** | 21/01/2008 | 12 | hla, hlb, hld, hlgB, clfA, clfB, fnbA, fnbB icaA, sdrC, sdrD | None |
| **1410111** | 21/01/2008 | 12 | hla, hlb, hld, hlgB, clfA, clfB, fnbA, fnbB icaA, sdrC, sdrD | None |
| **1410112** | 01/01/2008 | 16 | hla, hlb, hld, hlgB, clfA, clfB, fnbA, fnbB icaA, sdrC, sdrD | None |
| **1410113** | 01/01/2008 | 16 | hla, hlb, hld, hlgB, clfA, clfB, fnbA, fnbB icaA, sdrC, sdrD | None |
| **1410114** | 01/01/2008 | 16 | hla, hlb, hld, hlgB, clfA, clfB, fnbA, fnbB icaA, sdrC, sdrD | None |
| **1410115** | 21/01/2008 | 16 | hla, hlb, hld, hlgB, clfA, clfB, fnbA, fnbB icaA, sdrC, sdrD | None |
| **1410116** | 21/01/2008 | 16 | hla, hlb, hld, hlgB, clfA, clfB, fnbA, fnbB icaA, sdrC, sdrD | None |
| **1410117** | 21/01/2008 | 16 | hla, hlb, hld, hlgB, clfA, clfB, fnbA, fnbB icaA, sdrC, sdrD | None |
| **1410118** | 01/01/2008 | 18 | hla, hlb, hld, hlgB, clfA, clfB, fnbA, fnbB icaA, sdrC, sdrD | None |
| **1410119** | 28/01/2008 | 18 | hla, hlb, hld, hlgB, clfA, clfB, fnbA, fnbB icaA, sdrC, sdrD | None |
| **1410120** | 28/01/2008 | 18 | hla, hlb, hld, hlgB, clfA, clfB, fnbA, fnbB icaA, sdrC, sdrD | None |
| **1410121** | 28/01/2008 | 18 | hla, hlb, hld, hlgB, clfA, clfB, fnbA, fnbB icaA, sdrC, sdrD | None |
| **1410122** | 01/01/2008 | 19 | hla, hlb, hld, hlgB, clfA, clfB, fnbA, fnbB icaA, sdrC, sdrD | None |
| **1410123** | 01/01/2008 | 19 | hla, hlb, hld, hlgB, clfA, clfB, fnbA, fnbB icaA, sdrC, sdrD | None |
| **1410124** | 01/01/2008 | 19 | hla, hlb, hld, hlgB, clfA, clfB, fnbA, fnbB icaA, sdrC, sdrD | None |
| **1410125** | 21/01/2008 | 19 | hla, hlb, hld, hlgB, clfA, clfB, fnbA, fnbB icaA, sdrC, sdrD | None |
| **1410126** | 01/01/2008 | 20 | hla, hlb, hld, hlgB, clfA, clfB, fnbA, fnbB icaA, sdrC, sdrD | None |
| **1410127** | 01/01/2008 | 20 | hla, hlb, hld, hlgB, clfA, clfB, fnbA, fnbB icaA, sdrC, sdrD | None |
| **1410128** | 01/01/2008 | 20 | hla, hlb, hld, hlgB, clfA, clfB, fnbA, fnbB icaA, sdrC, sdrD | None |
| **1410129** | 06/01/2008 | 20 | hla, hlb, hld, hlgB, clfA, clfB, fnbA, fnbB icaA, sdrC, sdrD | None |
